# Sex Difference Impacts on the Relationship between Paraoxonase-1 (PON1) and Type 2 Diabetes

**DOI:** 10.3390/antiox9080683

**Published:** 2020-07-29

**Authors:** Valentina Rosta, Alessandro Trentini, Angelina Passaro, Giovanni Zuliani, Juana Maria Sanz, Cristina Bosi, Gloria Bonaccorsi, Tiziana Bellini, Carlo Cervellati

**Affiliations:** 1 Department of Biomedical and Specialist Surgical Sciences, Section of Medical Biochemistry, Molecular Biology and Genetics, University of Ferrara, 44121 Ferrara, Italy; v.rosta@ospfe.it (V.R.); tiziana.bellini@unife.it (T.B.); 2 Department of Morphology, Surgery and Experimental Medicine, University of Ferrara, 44121 Ferrara, Italy; giovanni.zuliani@unife.it (G.Z.); juana.sanz@unife.it (J.M.S.); cristina.bosi@unife.it (C.B.); gloria.bonaccorsi@unife.it (G.B.); carlo.cervellati@unife.it (C.C.); 3 Menopause and Osteoporosis Centre, University of Ferrara, 44124 Ferrara, Italy; 4 Center of Gender Medicine, University of Ferrara, 44121 Ferrara, Italy

**Keywords:** sex difference, paraoxonase-1, type-2 diabetes, oxidative stress, inflammation

## Abstract

Type-2 diabetes (T2D) and its cardiovascular complications are related to sex. Increasing evidence suggests that paraoxonase 1 (PON1) activity, an antioxidant enzyme bound to high-density lipoproteins (HDL), is implicated in the onset and clinical progression of T2D. Since we previously showed that PON1 is a sexual dimorphic protein, we now investigated whether sex might impact the relationship between PON1 and this chronic disease. To address this aim, we assessed PON1 activity in the sera of 778 patients, including controls (women, n = 383; men, n = 198) and diabetics (women, n = 79; men = 118). PON1 activity decreased in both women and men with T2D compared with controls (*p* < 0.05 and *p* > 0.001, respectively), but the change was 50% larger in the female cohort. In line with this result, the enzyme activity was associated with serum glucose level only in women (r = −0.160, *p* = 0.002). Notably, only within this gender category, lower PON1 activity was independently associated with increased odds of being diabetic (odds ratio (95% Confidence interval: 2.162 (1.075–5.678)). In conclusion, our study suggests that PON1-deficiency in T2D is a gender-specific phenomenon, with women being more affected than men. This could contribute to the partial loss of female cardiovascular advantage associated with T2D.

## 1. Introduction

Increasing evidence suggests that type-2 diabetes (T2D) and its macro- and micro-vascular complications are influenced by gender [1,2]. In particular, although women without T2D present a lower risk of cardiovascular diseases (CVDs) than men, the impairment in glucose metabolism seems to reverse this phenomenon [1,3,4]. The impact of gender on cardiometabolic factors leading to T2D cardiovascular complications could account for this epidemiological evidence [3,5].

Lipid abnormalities are important features in patients with T2D, who typically present high triglycerides and low high-density lipoprotein cholesterol (HDL-c) [6], thus contributing as main morbidity and mortality factors. Since it is well-known that this lipid pattern is similar in diabetic men and women [7], it cannot be the cause of the aforementioned sex-difference in T2D complications. Thus, other parameters should be considered to explain this epidemiological data. 

One of the best candidates in this frame is high-density lipoprotein (HDL) functionality. Several lines of evidence show that the sole cholesterol content of HDL particles does not fully capture their atheroprotective functions [8,9]. HDL has been ascribed with many beneficial activities. For instance, it has been shown to possess antioxidant, anti-inflammatory, and endothelial cell maintenance functions as well as playing a role in mediating reverse cholesterol transport [10,11,12]. These beneficial aspects of HDL are impaired in T2D, which significantly increases the risk of developing atherosclerosis and related diseases [13].

Paraoxonase 1 (PON1) is an accessory protein of HDL which, in intimate and mutual coordination with Apolipoprotein A1 (APO A1), contributes to the lipoprotein functionality [14,15,16]. In particular, it has been widely demonstrated that PON1 protects HDL, low-density lipoprotein (LDL), endothelial cells, and intimal macrophages from oxidative insult [17,18]. This protective action occurs via hydrolysis of the potential physiological substrate, lipo-lactones, which originate from the oxidative damage of phospholipids present on cell and lipoprotein surfaces [19,20]. Lipoprotein oxidation is central to the development of macro- (atherosclerosis) and micro-vascular disease in diabetes, as highlighted by the toxicity of oxidized lipoproteins for endothelial cells and pericytes in retinal capillaries [21,22]. Some studies report that PON1 activity is greater in women than men, this could be ascribed to an influential effect of sex hormones on the expression of the protein [23,24]. Indeed, in vivo studies found that mice treated with a male-pattern growth hormone had decreased hepatic mRNA levels of PON1 [25], whereas estrogens increased the stabilization/regulation of PON1 without affecting its expression [26]. Nonetheless, the evidence of differential expression of PON1 in the context of T2D is still undisclosed. Indeed, the only known information about PON1 expression in T2D is related to its genetic polymorphisms, which have been suggested to be responsible for variations in serum PON1 activity of T2D patients [27]. Consistently, the majority of population-based studies on the PON1 phenotype, found that low enzymatic activity is associated with T2D and related clinical complications, and this, in turn, could be due to the increased levels of pro-atherogenic oxidized LDL and HDL of these patients [28,29,30,31].

Recently, we have shown that sex significantly affects the interplay of PON1 with CVD risk factors, such as overall and central obesity [23]. In this study, we investigated in a large population of men and women whether the effect of gender on PON1 might contribute to the sexual dimorphism that characterizes complicated T2D. 

## 2. Materials and Methods 

### 2.1. Subjects

In the present study, we re-examined the data collected from three different cohorts: (1) subjects attending the metabolic outpatient clinic of Sant’Anna University Hospital (Ferrara, Italy); (2) outpatients undergoing bone densitometry testing at the Menopause and Osteoporosis Centre of the University of Ferrara; (3) outpatients referring to the Obesity Centre of Sant’Anna (more details in [32,33,34].

The research protocols conform to The Code of Ethics of the World Medical Association (Declaration of Helsinki) and were conducted accordingly to the guidelines for Good Clinical Practice (European Medicines Agency). The studies were approved by the Local Ethics Committee of the University of Ferrara, and written informed consent was obtained from each patient during the first office visit before the possible inclusion in the study. No personal information was available to the authors of the study to protect the anonymity of the patients. 

Inclusion criteria were men/women older than 18 years. Exclusion criteria were infection, acute or chronic disease (affecting liver, kidney, lungs, etc.), cancer, dementia, pregnancy, and alcohol consumption >10 g daily. 

The diagnosis of T2D was made by following the American Diabetes Association (ADA) criteria.

Accordingly, a total of 778 subjects were divided into unaffected Controls (n = 581) and T2D subjects (n = 197).

At study entry, clinical (a questionnaire about comorbidities, lifestyle, and blood pressure) and anthropometric data (i.e., body mass index, BMI) were collected from each patient (however, anthropometric data were available only for a part of the sample subjects). Glucose level was assayed in all samples by the Central laboratory of S. Anna University Hospital, Ferrara, whereas plasma lipid profile (including total cholesterol, triglycerides, HDL-c, and LDL-c) was retrieved only for a part of the whole population (total, n = 685; controls, n = 545; T2D, n = 140).

### 2.2. Serum Sampling and Biochemical Assays

Venous blood samples from patients were collected after overnight fasting and centrifuged at 1500*g* for 10 min. The serum was aliquoted and stored at −80° C until analysis.

All assays were performed by UV-VIS spectrophotometric techniques in a 96-well plate format by using a Tecan Infinite M200 microplate reader (Tecan group Ltd.).

Arylesterase activity was assayed by adding 10 μL of serum to 240 μL of reaction mixture consisting of 1 mmol/L phenylacetate and 0.9 mmol/L CaCl_2_ dissolved in 9 mmol/L Tris-HCl, pH 8, according to previously published methods [33]. A molar extinction coefficient of 1.3 × 10^3^ L^−1^ mol^−1^ cm^−1^ was used for the calculation of enzyme activity, expressed in kilo unit per liter. One unit of arylesterase activity accounts for 1 µmol of phenol produced in a min under the conditions of the assay. The intra-assay Coefficient of Variation (CV) was 3.8% whereas the inter-assay CV was 9.7%.

Total cholesterol (Tc), HDL-c, triglycerides, and glucose were assayed by routine enzymatic-colorimetric methods and LDL-c was calculated according to the Friedewald formula.

### 2.3. Statistical Analysis

Continuous variables were first analyzed for normal distribution using the Kolmogorov–Smirnov tests. Since the variables were not normally distributed, group comparisons were performed using the Mann–Whitney U test or t-test on the square root transformed variable (to approach a normal distribution). A chi-squared test was used to compare categorical variables between groups. Bivariate correlation analyses were performed using Spearman’s tests. Multiple regression analysis was used to determine the independence of the found associations involving arylesterase in men and women. For all the models, residuals were visually inspected and tested for normality. Multivariate logistic regression analysis was performed to evaluate the association between low levels of PON-arylesterase activity and the risk of T2D. To this end, PON-arylesterase activity values in the whole population were divided into quartiles based on the distribution data of the control group. The outcome variable was the absence/presence of diabetes, whereas PON-arylesterase organized into quartiles was the predictor, with the highest quartile considered as the reference category. To correct for confounding factors and test the independency of predictor, we included age centered on the mean (64.72 years), smoking habits, hypertension, and HDL-c as covariates. These two analyses were performed on men and women separately. The multinomial logistic regression was also performed on the PON-arylesterase continuous variable, after centering for the mean in the whole population (91.6004 kU/L) by evaluating the interaction term sex*PON-arylesterase activity as the main clue for the influence of both PON-arylesterase and sex on the outcome. Both uncorrected and corrected multivariable logistic regression (for age, smoking habits, hypertension, and HDL-c) were performed. In all the analyses, a *p* < 0.05 was considered statistically significant.

## 3. Results

### 3.1. Population Characteristics

The demographic and clinical characteristics of the whole population and grouped according to sex and disease status are summarized in Table 1.

As expected, subjects with T2D were characterized by a general worst lipid profile (high triglycerides, and low HDL-c, *p* < 0.001) than controls, although they demonstrated slightly lower total cholesterol and LDL-C than controls (*p* < 0.001). On the contrary, comorbidities such as hypertension and CVDs where more frequent in diabetic subjects (Table 1, *p* < 0.001 for both comorbidities). The differences observed in the lipid profile and comorbidities prevalence were grossly conserved when men and women were considered separately (for the complete results, see Table 1).

### 3.2. PON-Arylesterase in Diabetic and Non-Diabetic Women and Men

**Figure 1:**
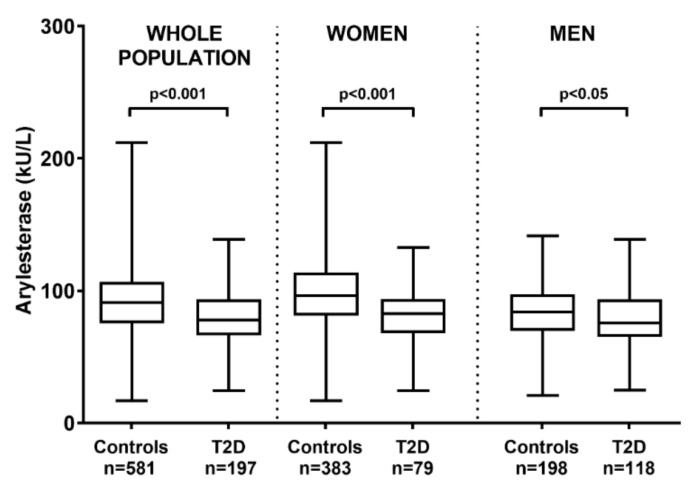
PON-arylesterase activity measured in the whole population, or subjects grouped in women and men. As depicted, we found significantly lower levels of PON-arylesterase activity in subjects affected by T2D compared with controls (*p* < 0.001), the difference was maintained also within sexes (*p* < 0.001 and *p* < 0.05, for women and men, respectively). Mann–Whitney U test was used for comparisons. PON: paraoxonase-1; T2D: Type 2 diabetes.

Figure 1 displays PON-arylesterase activity levels in controls and T2D in the whole population, and separately in women and men. 

Diabetic subjects showed a decreased activity of PON1 when compared with controls (*p* < 0.001, effect size r = 0.24). The same difference, although with a different magnitude, was also observed in the two subsets of women and men (*p* < 0.001, effect size r = 0.25, and *p* < 0.05, effect size r = 0.11, respectively). Of note, these differences remained significant even by comparing the square root transformed variable in T2D subjects and controls with a t-test (whole population, *p* = 0.001, effect size r = 0.24; women, *p* = 0.001, effect size r = 0.25), with the exception of men which did not show any difference (*p* = 0.082, effect size r = 0.1).

In agreement with the apparent link between T2D and low PON1, we found an inverse correlation between serum glucose levels and PON1 activity in the whole population (r = −0.209, *p* < 0.01). Notably, this association was significant in women but not in men (r = −0.162, *p* < 0.01 and r = −0.099 *p* = 0.125, respectively) (see Figure S1).

### 3.3. Logistic Regression Analysis for the Association of PON1 Decrease and T2D in Relation to Sex

We further explored the possible influence of sex on the association between PON1 activity and T2D by employing a logistic regression analysis. 

The analysis was performed on the whole population without grouping by sex and by treating PON-arylesterase activity as a continuous predictor, presence/absence of T2D as an outcome, and by evaluating both the main effect of sex and the sex* PON–arylesterase interaction as clues for the influence of sex on the outcome. As summarized in Table S1 (Model 1), PON-arylesterase activity was a significant predictor associated with T2D development, since an increase in one unit of its activity caused a decrease in the odds of being diagnosed with T2D (Odds Ratio, O.R., (95% Confidence Interval, 95%CI): 0.97 (0.96–0.98)). Sex was confirmed to be a strong risk factor for the disease, with men characterized by greater odds of being affected by T2D (O.R.: 2.55 (1.77–3.67)). In addition, PON-arylesterase and sex seem to interact given the significant interaction coefficient we observed in Model 1 (see Table S1). Upon correction for possible confounders like age, smoking habits, hypertension, HDL-c (see Table S1, Model 2), both the main effects (PON-arylesterase and sex) and the interaction (O.R.: 1.025 (1.002–1.045), *p* = 0.010) remained significant.

**Figure 2:**
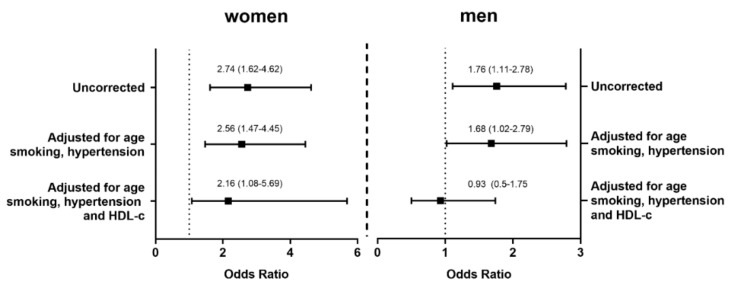
Odds ratios (95% C.I.) for the diagnosis of T2D in women and men with low levels (lower than IV quartile) of serum PON-arylesterase activity. Women with low levels of PON-arylesterase activity demonstrated increased odds of being affected by T2D independently from all cofounding factors (left panel). On the contrary, while the uncorrected model and the model corrected for age, smoking and hypertension suggested increased odds for diabetes in men as well, the inclusion of HDL-c as covariate made the relationship non-significant. HDL-c: High Density Lipoprotein-cholesterol; T2D: Type 2 Diabetes.

We further explored the association between sex and PON-arylesterase in T2D by dividing the activity into quartiles based on the distribution in the control group, and by comparing the odds of being diagnosed with T2D of subjects with activity lower than < 110.9 kU/L (the IV quartile cut-off) with those within the higher activity quartile (>110.9 kU/L). As displayed in Figure 2, both men and women with PON-arylesterase levels lower than the highest quartile had greater odds of being affected by T2D (Women: O.R. (95% Confidence Interval): 2.74 (1.62–4.62); Men: 1.76 (1.11–2.78)). These associations became weaker but remained significant after adjusting for age, smoking habits, and hypertension (Women: O.R. (95% Confidence Interval): 2.56 (1.47–4.45); Men 1.68 (1.02–2.79)). Adding HDL-c to the covariate set caused a further decrease in the strength of the relationship, in particular in men, where low PON1 activity was no more related to high odds of being affected by T2D (Figure 2, right panel).

**Figure 3:**
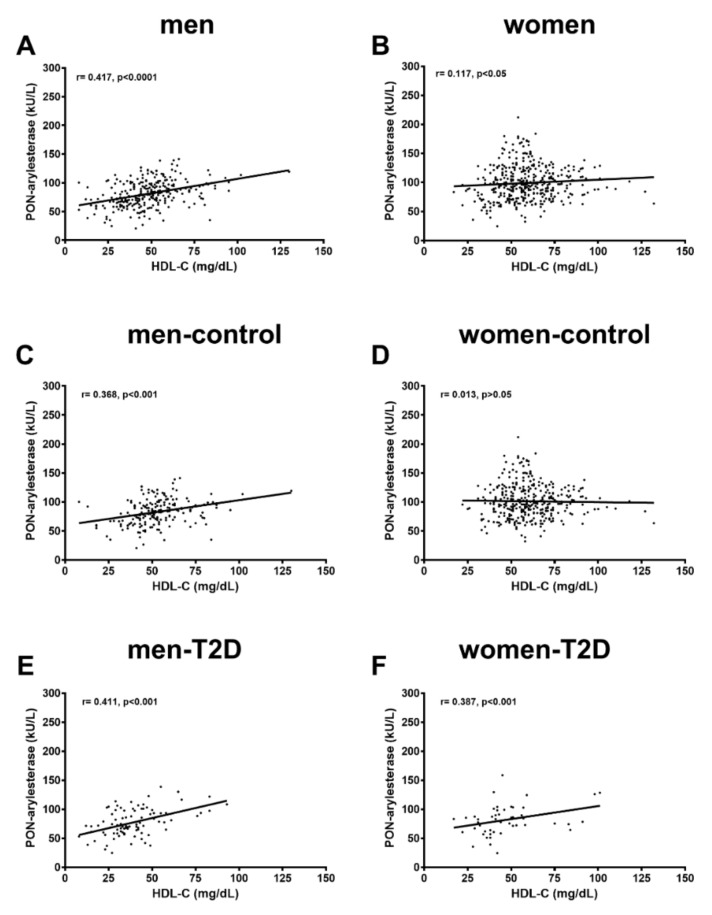
Correlations between PON-arylesterase activity and HDL-C levels in men (**A**) and women (**B**) and separated according to diagnosis in control men (**C**), men with T2D (**E**), control women (**D**) and women with T2D (**F**). As displayed, the correlation we found was mainly driven by men in both control and diabetes groups.

The observation that this effect was much more relevant in men than in women was likely due to the different strengths in the correlation between PON1 and HDL-c within these two groups (Figure 3). Indeed, we found that the association in the whole population sample (r = 0.317, *p* < 0.001) was mostly driven by men (Figure 3, men: r = 0.417, *p* < 0.0001, women: r = 0.117, *p* < 0.05). Of note, when subjects were divided according to diagnosis of diabetes, only T2D women (r = 0.387, *p* < 0.01) and men in both control and T2D groups demonstrated a significant positive association between PON-arylesterase activity and HDL-c (r = 0.368, *p* < 0.001 and r = 0.411, *p* < 0.001, respectively).

## 4. Discussion

The present study confirms and extends previous findings on the implication of PON1 in T2D, but also demonstrated for the first time that sex significantly affects the interplay between the antioxidant enzyme and this disease. Indeed, we found that PON1 was decreased in women and, to a lesser extent, in men, but only in women was the difference independent of confounders such as age, smoking status, hypertension, and HDL-c. On the contrary, the change in men appeared to merely reflect the decrease in the T2D-related HDL level. Of note, the calculated effect sizes, a measure of the magnitude of the association between two variables (in this case PON1 and T2D), suggest that the effect of sex on PON1 in the context of T2D is small/moderate but significant (r = 0.25 of women and r = 0.11 for men; according to the classical definition from Cohen, r = 0.1, 0.3, or 0.5 indicates a small, medium, or large effect size, respectively).

In line with other studies [35,36,37], we found that PON1 activity is significantly lower in patients with T2D compared to controls. Many of the published investigations on this topic are characterized by a relatively small and medium sample size. A major disadvantage of these studies is that the confidence intervals of the observed relationships are very broad and therefore lack the precision to quantify involvement of PON1 in the disease development. Our study is, to the best of our knowledge, the largest one on this research topic.

Mounting evidence suggests a scenario where PON1 and T2D are reciprocally related [28,29,30,31,32,33,34,35,36,37]. Various factors contribute to the development of T2D, such as genetic, lifestyle, and environment [24,25,26,27,28,29,30,31,32,33,34,35,36,37,38,39]. The genetic predisposition of having low PON1 expression and activity has been ascribed as one of these influential factors by several authors [24]. The most studied functional single-nucleotide polymorphisms (SNPs) in the *PON1* gene are Q192R and L55M. A recent meta-analysis showed that these SNPs Q192R/L55M are significantly associated with the risk of T2D [40]. In vitro studies suggest that PON1 could participate in T2D pathogenesis by protecting against glucose inflammation and oxidative stress (OxS) cytotoxicity and may play a role in the secretion of insulin from these cells [41,42]. On the other hand, reduction in PON1 activity could also represent an effect of oxidative and glycation stress associated with T2D. Indeed, it has been shown that these challenges reduce the catalytic efficiency of the enzyme in a dose-dependent manner [43]. However, the evidence of sex-influence of PON1 expression in the context of T2D is still lacking, and limited to animal studies evaluating only non-diabetic females and males [25,26].

Independently of the causality of PON1 in T2D, the published evidence is concordant on the fact that the contribution of the protein to the beneficial properties of HDL is compromised in diseased patients [28]. This PON1 deficiency reflects in a weaker anti-atherogenic action, with HDL from T2D patients being less effective in counteracting the inhibitory effect of oxidized LDL on vasorelaxation [44] and endothelial nitric oxide synthesis [45]. In line with this reasoning, reduced PON1 activity has been linked to a high risk of T2D-related CVD complications in several studies [29,35,46].

However, our data suggest that this decrease in enzyme activity is not homogeneously distributed in the diabetic population, but it might involve mostly the female part. Indeed, in women, but not in men, the presence of T2D appears to be associated with two independent adverse phenomena such as decreased HDL-c concentration (a typical feature of diabetic dyslipidemia) and function. This would suggest that T2D attenuates the reported “double” physiological female sex advantage represented by the high HDL-c concentration and PON1 activity level [1,47,48]. As previously postulated by us and others, high levels of this enzyme might contribute to the lower risk of CVD in women compared to men [23,24,49]. Thus, the sex-dependent decrease in PON1 could be one of many biological factors explaining why T2D increases the risk for CVD in females to a greater extent than in males (see comprehensive review on sex difference in T2D complications and comorbidities [1,2,3]). Notably, the logistic regression outcomes suggest that only in women the decrease in PON1 activity due to T2D is independent of HDL-c. The strong confounding effect of HDL-c we observed is not surprising since this marker is a surrogate for HDL particle concentration which, in turn, is the main carrier of PON1 [14,50].

However, the observational nature of our study makes challenging to speculate on the biological mechanisms and factors underlying the apparent sex-dependent change of PON1 activity in T2D patients. Nevertheless, some intriguing clues in this regard are provided by the analysis of the correlation between HDL-c (i.e., surrogate maker of HDL particle) and PON1. Indeed, we found that the correlation between the enzyme activity and the level of its main carrier in circulation is much stronger in men relative to women, and it disappeared when non-diabetic women were considered separately. This difference might be connected to the already demonstrated shift of the accessory protein from an HDL subtype to another when diabetes occurs [51], which could also impact the catalytic efficiency of the protein. HDL belongs to a highly heterogeneous family which is composed of particles differing to each other for size, density, lipid, and protein constituents. Contrary to men, in non-diabetic women, PON1 could be segregated in one subtype in healthy women (e.g., the small HDL_3_ or the buoyant HDL_2_), and this might explain the lack of association with the whole HDL-c level. Then, with the occurrence of diabetes and the related oxidation/glycation of the lipoprotein PON1 could redistribute in other subclasses.

This study was not without limitations. First, the cross-sectional design precluded the ability to determine with certainty any cause–effect relationship between PON1 activity and the increased susceptibility of women to T2D compared to men. Second, potential confounding factors not considered in the study and the unequal size of sample groups might have affected the reliability of the results. Despite these undeniable caveats, this is, to the best of our knowledge, the largest population-based study on this topic. 

In conclusion, our study suggests that PON1-decreased activity in T2D is a gender-specific phenomenon, with women being more affected than men. This difference might, at least partially, explain why diabetic women are more prone to develop cardiovascular complications than diabetic men. The decrease in PON1, reflecting in a poorer HDL functional quality, could represent a target for nutraceutical or pharmacological treatment in both sexes, but in particular in women. More in general, our findings strengthen the concept that since many diseases can manifest differently in men and women, searching for biological factors underlying this diversity becomes of crucial importance for defining the medical strategy.

## Figures and Tables

**Table 1:** Main clinical and demographic characteristics of controls and diabetic subjects in the whole population and divided by sex.

	Whole Population		Women		Men	
	Controls (n = 581)	T2D (n = 197)	Controls (n = 383)	Diabetes (n = 79)	Controls (n = 198)	Diabetes (n = 118)
Age, years	64 ± 12	65 ± 10	63 ± 11	64 ± 11	67 ± 11	67 ± 10
Hypertension, n (%)	233 (40)	161 (81) ^a^	138 (36)	62 (72) ^a^	95 (48)	99 (84) ^a^
CVD, n (%)	29 (5)	56 (28) ^a^	10 (3)	20 (25) ^a^	18 (9)	36 (31) ^a^
Smoking, n (%)	121 (21)	29 (15)	88 (23)	15 (19)	45 (23)	13 (11) ^b^
Glucose, mg/dL *	95 ± 11	141 ± 38 ^a^	93 ± 10	129 ± 35	98 ± 10 ^a^	146 ± 38 ^a^
Lipid lowering drugs ^§^	127 (20)	130 (66) ^a^	107 (28)	39 (50) ^a^	20 (10)	91 (77) ^a^
Lipid profile ^#^						
HDL-c, mg/dL	56 (47–67)	40 (31–65) ^a^	58 (50–69)	42 (38–56) ^a^	52 (44–60)	37 (30–47) ^a^
LDL-C, mg/dL	132 ± 35	113 ± 42 ^a^	136 ± 32	126 ± 46 ^b^	124 ± 38	106 ± 37 ^a^
Triglycerides, mg/dL	91 (69–129)	148 (103–215) ^a^	90 (70–123)	147 (112–203) ^a^	92 (68–119)	149 (99–225) ^a^
Total cholesterol, mg/dL	210 ± 40	190 ± 45 ^a^	217 ± 37	208 ± 46	194 ± 41	180 ± 41 ^a^

Continue variables are expressed as mean ± standard deviation (SD) or median (interquartile range). Categorical variables are expressed as frequency (percentage). T2D: Type-2 diabetes; CVD: cardiovascular disease; HDL-c: High-density lipoprotein cholesterol; LDL-c: Low-density lipoprotein cholesterol; PON; paraoxonase-1. ^a^ *p* < 0.001 vs. controls. ^b^ *p* < 0.05 vs. controls.* Glucose level was available for 634 subjects. ^§^ Information regarding the use of lipid-lowering drugs was available for 602 subjects. ^#^ Complete lipid profile was available for 685 subjects.

## Supplementary Materials

The following are available online at https://www.mdpi.com/2076-3921/9/8/683/s1, Figure S1: Correlations between glucose and PON1-arylesterase activity, Table S1: Logistic regression analysis for the association between PON-arylesterase activity, T2D, and sex.
