# Supplementary material for: Sex Difference Impacts on the Relationship between Paraoxonase-1 (PON1) and Type 2 Diabetes
Source: Antioxidants (Basel). 2020 Jul 29;9(8):683. doi: 10.3390/antiox9080683 (PMC7463677; doi:10.3390/antiox9080683)
Supplement: Supplementary file 1 [file antioxidants-09-00683-s001.zip › Supplementary tables_22.05.2020.docx]

**Supplementary Results**

| **Supplementary Table 1.** Logistic regression analysis for the association between PON-arylesterase activity, T2D and sex. | | | |
| --- | --- | --- | --- |
| **Model 1** | **B (SE)** | **O.R. (95% CI)** | **P-value for the coefficient** |
| PON-Arylesterase (centered) | *-0.027 (0.005)* | *0.97 (0.96-0.98)* | *<0.0001* |
| Sex (male=1) | *0.935 (0.186)* | *2.55 (1.77-3.67)* | *<0.0001* |
| PON-Arylesterase*Sex | *0.017 (0.008)* | *1.018 (1.002-1.033)* | *0.024* |
| Constant | -1.56 (0.13) | 0.21 |  |
| **Model 2** |  | | |
| PON-Arylesterase (centered) | *-0.018 (0.007)* | *0.98 (0.96-0.99)* | *0.012* |
| Sex (male=1) | *0.660 (0.246)* | *1.94 (1.19-3.14)* | *0.007* |
| PON-Arylesterase*Sex | *0.025 (0.010)* | *1.025 (1.006-1.045)* | *0.010* |
| Age (centered) | *-0.023 (0.011)* | *1.024 (1.002-1.045)* | *0.029* |
| Smoking habits (yes) | -0.563 (0.303) | 0.569 (0.314-1.032) | 0.063 |
| Hypertension (yes) | *1.311 (0.263)* | *3.71 (2.22-6.21)* | *<0.0001* |
| HDL-c | *-0.061 (0.009)* | *0.941 (0.924-0.958)* | *<0.0001* |
| Constant | 0.591 (0.545) | 1.805 |  |
| For Model 1, Cox&Snell R^2^=0.089, Nagelkerke R^2^=0.132. For Model 2, Cox&Snell R^2^=0.236, Nagelkerke R^2^=0.370. The significant relationships are highlighted in italic.  Model 1: uncorrected model; outcome: diabetes; predictors: PON-arylesterase, sex, PON-arylesterase*sex interaction; Model 2: Model 1 corrected for age, smoking habits, hypertension, CVDs.  B: regression coefficient; SE: standard error; O.R.: odds ratio; 95% CI: 95% Confidence Interval.  Mean age for centering = 64.72 years; mean value of PON-arylesterase for centering = 91.6004 kU/L.  ^a^p<0.05; ^b^p<0.01; ^c^p<0.001 | | | |
