# Supplementary material for: Sex Difference Impacts on the Relationship between Paraoxonase-1 (PON1) and Type 2 Diabetes
Source: Antioxidants (Basel). 2020 Jul 29;9(8):683. doi: 10.3390/antiox9080683 (PMC7463677; doi:10.3390/antiox9080683)
Supplement: Supplementary file 1 [file antioxidants-09-00683-s001.zip › Supplementary figures_22.05.2020.docx]

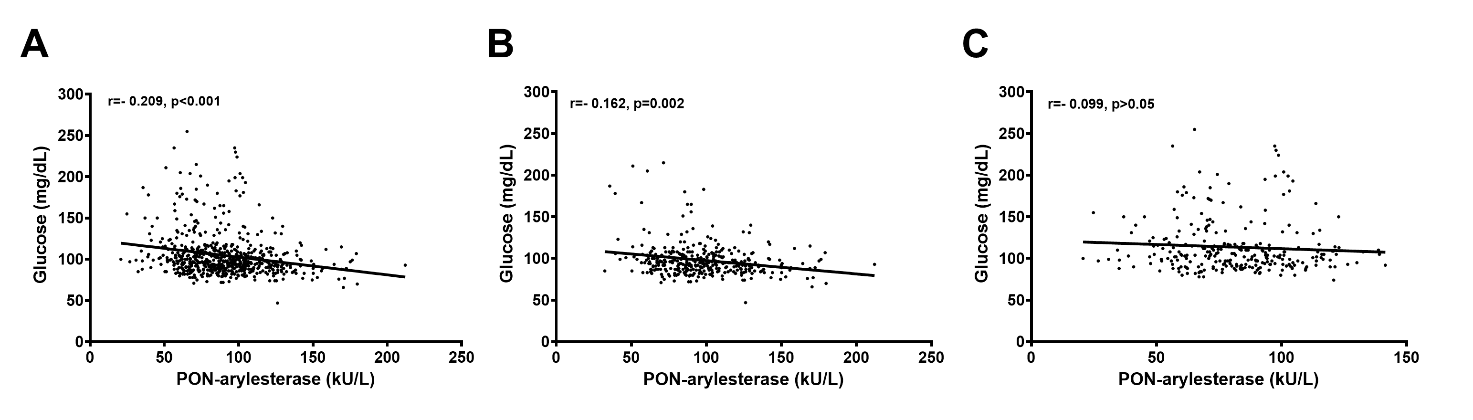


**Supplementary Figure 1.** Correlations between glucose and PON1-arylesterease activity in the whole population (panel A), in women (panel B) or in men (panel C). As reported, the correlation was significant and negative in the whole population and in women, but was not significant in men.
